# Supplementary material for: Organic matter composition and greenhouse gas production of thawing subsea permafrost in the Laptev Sea
Source: Nat Commun. 2022 Aug 27;13:5057. doi: 10.1038/s41467-022-32696-0 (PMC9420143; doi:10.1038/s41467-022-32696-0)
Supplement: Supplementary file 1 — Supplementary Information [file 41467_2022_32696_MOESM1_ESM.pdf]

Supplementary Material

Organic matter composition and greenhouse gas production of thawing subsea permafrost in the Laptev Sea

Birgit Wild, Natalia Shakhova, Oleg Dudarev, Alexey Ruban, Denis Kosmach, Vladimir Tumskoy, Tommaso Tesi, Hanna Joss, Inna Nybom, Felipe Matsubara, Helena Alexanderson, Martin Jakobsson, Alexey Mazurov, Igor Semiletov, Örjan Gustafsson

1. Supplementary Methods

1.2 Optically stimulated luminescence chronology

Samples for OSL dating were processed under dark room conditions. Subsamples of core 4D-12 were taken by pushing a tube into the drill core that contained frozen sediments. The tubes were opened at the Lund Luminescence Laboratory, Sweden. Samples were wet sieved to extract the 180-250 µm fraction (90-180 µm for one sample at 15 m depth due to its finer-grained nature) and subsequently treated with 10% HCl, 10% H_2O_2, 38% HF (60 min.) and a second time with 10% HCl. Density separation with LST Fastfloat at 2.62 g cm^{-3} was used to separate quartz from feldspar grains before HF etching. After the final HCl treatment, extracts were re-sieved at 180 µm (90 µm for one sample at 15 m depth).

Dose measurements were carried out on large aliquots in a Risø TL/OSL reader model DA-20 with a $^{90}Sr/^{90}Y$ beta radiation source (~0.15 Gy s^{-1}). Single Aliquot Regeneration protocol[1–4] settings were determined individually for each sample based on infrared/blue ratios, dose recovery and preheat plateau tests (Supplementary Table 1). Most samples suffered from some apparent feldspar contamination (high infrared/blue ratios) and had relatively dim signals and significant scatter in equivalent doses. A fast signal component was nevertheless present and dominating, and dose recovery tests showed that the analytical protocols gave accurate results (mean ratio 0.97 ± 0.05, n = 15). However, two samples, from 35 and 47 m depth, were rejected due to very poor dose recovery ratios and not measured further (Supplementary Table 1). Aliquots were accepted if they had a recycling ratio <10% from unity and a test dose error <10% (<20%

for samples at 17, 42, and 51 m depth). Equivalent doses were calculated in Risø Analyst, using exponential curve fitting, and the Central Age Model[5] was applied.

The sediment dose rate was determined by gamma spectrometry at the Nordic Laboratory for Luminescence Dating in Denmark[6]. Field and saturated water content were measured by weighing subsamples in the laboratory, and the average water content since time of deposition was estimated as having been saturated for 90% of the time and unsaturated (water content as when opened in the laboratory) during 10% of the time. Environmental dose rates and final ages were calculated in the DRAC online calculator[7].

1.2 Lignin biomarker analysis

Micro-wave assisted CuO oxidation of freeze-dried and ground samples was used to hydrolyze the macromolecules that constitute the bulk of organic matter, and analyze the derived lignin phenols, hydroxybenzenes and *p*-hydroxybenzenes[8]. Sample aliquots were amended with CuO, ferrous ammonium sulfate, and NaOH and oxidized in O_2-free atmosphere in an UltraWAVE Milestone microwave. After oxidation, we added known amounts of ethyl vanillin and *trans*-cinnamic acid as internal standards, acidified samples with HCl to pH 1 and extracted twice with ethyl acetate. Excess water was removed with anhydrous Na_2SO_4, and the solvent was evaporated in a centrifugal evaporator under reduced pressure at 60°C. Samples were dissolved in pyridine and stored frozen until analysis, and then derivatized with bis-trimethylsilyl-trifluoroacetamide (BSTFA) + 1% trimethylchlorosilane (TMCS). The CuO oxidation products were analyzed with a gas chromatography-mass spectrometry (GC-MS) system (Agilent 7693 autosampler, 7820A GC, 5977E MSD) with splitless injection on a DB1-MS column (30 m x 250 µm; 0.25 µm film thickness), with 1.2 ml min^{-1} helium as carrier, an initial temperature of 50°C for 5 min, a ramp of 10°C min^{-1} to 300°C, and constant temperature for 8 min. We quantified vanillin (Vl), acetovanillone (Vn), vanillic acid (Vd), syringaldehyde (Sl), acetosyringone (Sn), syringic acid (Sd), *p*-coumaric acid (*p*Cd), ferulic acid (Fd), benzoic acid (Bd), *m*-hydroxybenzoic acid (*m*-Bd), 3,5-dihydroxybenzoic acid (3,5-Bd), *p*-hydroxybenzaldehyde (Pl), *p*-hydroxyacetophenone (Pn), and *p*-hydroxybenzoic acid (Pd) against

external standard curves of each compound that were measured together with the samples. Individual values are presented in Supplementary Table 9.

1.3 Lipid biomarker analysis

Lipid biomarkers were extracted from freeze dried samples with accelerated solvent extraction (ASE; Dionex ASE 350). Sediment samples were packed in 34 ml ASE cells with pre-combusted glass fiber filters and glass wool, amended with internal recovery standards (d50-tetracosane, d62-triacontane, d39-eicosanoic acid, 2-hexadecanol, and d5-stigmasterol), and extracted with dichloromethane:methanol (9:1, v/v), with three extraction cycles at 85 °C (static time 5 min, pressure 100 bar, flush volume 60%, purge time 1 min). Extracts were treated with acid-activated copper and anhydrous sodium sulfate (pre-combusted 450°C, 4 h) and allowed to react at room temperature overnight, for removal of sulfur and water.

Solvent was reduced in volume with a rotary evaporator, followed by fractionation and purification as previously described[9]. Shortly, BondElut cartridges (Agilent Technologies, USA) were used to separate the neutral fraction and *n*-alkanoic acids by eluting with 15 ml of dichloromethane: isopropanol (2:1) and 15 ml of diethyl ether with 2% acetic acid. The neutral fraction was separated into a non-polar (containing *n*-alkanes) and a polar fraction (containing *n*-alkanols and steroids), using Al_2O_3 columns (0.5 g, 100% activated 450°C for 4 h) packed in a glass pipette, by eluting with 3 ml hexane:dichloromethane (9:1), and 3 ml of methanol:dichloromethane (1:1). All fractions were reduced in volume under gentle N_2 flow, and solvents were exchanged to *n*-hexane. Injection standard (*p*-terphenyl) was added prior analysis, and the *n*-alkanoic acid and polar fractions were derivatized with BSTFA + 1% TMCS (80°C, 60 min). Samples were analyzed with GC-MS (see above) on a DB-5 column (30 m × 250 µm; 0.25 µm film thickness), with the temperature program starting at 60°C, a gradient of 10°C per minute until 310°C, and a hold time of 16 min. Concentrations of individual *n*-alkanes, *n*-alkanoic acids, *n*-alkanols and steroids were quantified against external standards. Limits of Detection (LOD) were determined as blank average plus three times standard deviation. All samples were corrected for average blank levels and recoveries. The recoveries of the internal standards were 87 ± 5% for *n*-alkanes, 75 ± 26% for *n*-alkanols and 53 ± 22% for *n*-alkanoic acids.

Individual values on *n*-alkanes, *n*-alkanoic acids and *n*-alkanols are presented in Supplementary Tables 10-12.

1.4 Incubation experiment

The incubation experiment was designed to measure production rates of CH_4, CO_2 and N_2O by decomposition of thawed subsea permafrost organic matter under conditions as close as possible to the natural environment (i.e., cold and anoxic), and to determine the ^{13}C- and D-isotopic composition of produced CH_4. One sample from each core was chosen randomly for the incubation experiment, covering a range of depths: 4D-13 at 4.55 m depth, 4D-12 at 27.4 m, 2D-13 at 24.7 m and 1D-14 at 25.1 m. Frozen subsea permafrost material was sub-sampled at -16°C and aliquots of ca. 12 g wet weight placed in 100 ml GL45 screw-cap flasks equipped with O_2 sensor stickers to monitor anoxic conditions (PreSens SP-PSt6-NAU-D5-YOP). Aliquots of 5 g wet weight were additionally sub-sampled, and dried at 60°C to determine water content. Flasks were moved to a glove box (Coy Polymer anaerobic chamber) with anoxic atmosphere at room temperature for further processing, minimizing exposure of thawing samples to oxic conditions. Samples were amended with 1.5 ml de-gassed Milli Q water before flasks were closed with butyl rubber stoppers (Glasgerätebau Ochs; pre-boiled to minimize O_2) and hole-caps. Flasks were flushed with 0.5 bar N_2 for 15 minutes, and moved from the glove box to an incubator (Memmert ICP110) at 4°C (compared to the *in-situ* range of -1 to +1°C in unfrozen sediments). Three empty flasks were processed in the same way as blanks.

Concentrations of greenhouse gases were determined on days 1, 5, 9, 16, 23, 37, 114, 286, and 601 of the incubation. Headspace volumes of 7 ml were removed inside the glove box; the removed headspace gas was then replaced by the same volume of N_2. Headspace samples were analyzed for concentrations of CH_4, CO_2 and N_2O on an SRI 8610C GC with flame ionization and electron capture detectors on days 1-114 and 601, and for concentrations of CH_4 and CO_2 on an Agilent 7890A GC with flame ionization detector on day 286. Concentrations were calibrated against standard gases at three concentration levels and production rates

were calculated, accounting for the removal and replacement of headspace gas. Data on greenhouse gas production rates at individual time point are presented in Supplementary Table 5.

The isotopic composition of accumulated CH_4 was analyzed after 335 days of incubation. Two headspace samples with 2.1-2.7 ppm CH_4 were taken from each sample, and aliquots of 25 ml were injected in pre-evacuated 20 ml headspace vials and shipped to the Institute for Marine and Atmospheric Research at Utrecht University. Sampled gas was flushed with zero air into the extraction system as described previously[10]; an Ascarite/$Mg(ClO_4)_2$ trap was installed to remove the CO_2 before the CH_4 was trapped. The CH_4 isotopic composition was analyzed on a Thermo Scientific Delta V IRMS. The δD and $\delta^{13}C$ values of CH_4 were calculated using a one-point calibration, resulting in a precision of ± 3‰ for δD and ± 0.1‰ for $\delta^{13}C$. Individual data are presented in Supplementary Table 7. Incubation flasks were flushed with N_2 after removing large volumes for isotopic analysis to re-set the headspace.

The development of CO_2 and CH_4 production over time was described by fitting two-pool models[11] for each sample following Equ. 1.

$$Cumulative\ CO_2\ or\ CH_4 = C_L \times \left(1 - e^{(-k_L \times time)}\right) + k_R \times time \qquad \text{(Equ. 1)}$$

C_L and k_L represent the size and mineralization constant of the “labile”, i.e., faster-degrading, organic carbon pool, and k_R the mineralization constant of the “recalcitrant”, slower-degrading pool (but see the discussion for interpretation). The initial lag period before onset of CH_4 production was removed before fitting the CH_4 models. Model parameters were calculated using the nls function in R.

Supplementary Table 1. Optically stimulated luminescence ages and supporting data for the subsea permafrost drill core 4D-12. All samples were measured with preheat 220°C and cut heat at 200°C. The Central Age Model[5] was used to calculate the dose. Samples Lund-12063 and Lund-12065 were rejected due to very poor dose recovery ratios.

Lab no.	Sample no.	Depth	Age	Dose	n	Dose rate	Water	Stimulation[a]	Dose recovery ratio	Note
		m	ka	Gy	acc./total	Gy ka^{-1}	%			
Lund-12059	IVD-2012-1	15	8.52 ± 0.59	25.8 ± 1.3	21/24	3.02 ± 0.15	40	Blue	0.97 ± 0.02	b
Lund-12060	IVD-2012-2	17	50.8 ± 5.0	128 ± 11	18/24	2.52 ± 0.13	36	Pulsed	1.03 ± 0.07	c
Lund-12061	IVD-2012-3	24	70.4 ± 6.2	226 ± 16	15/19	3.21 ± 0.17	20	Post-IR blue	1.00 ± 0.14	d
Lund-12062	IVD-2012-4	28	75.2 ± 5.5	233 ± 13	20/21	3.09 ± 0.15	17	Post-IR blue	0.90 ± 0.17	b
Lund-12063	IVD-2012-5	35	Rejected			2.90 ± 0.12	16	Post-IR blue	0.62 ± 0.09	
Lund-12064	IVD-2012-6	42	141 ± 19	331 ± 40	16/21	2.34 ± 0.13	22	Pulsed	0.96 ± 0.16	c
Lund-12065	IVD-2012-7	47	Rejected			2.28 ± 0.10	26	Pulsed	1.38 ± 0.17	
Lund-12066	IVD-2012-8	51	162 ± 22	257 ± 31	16/30	1.59 ± 0.11	29	Post-IR blue	0.94 ± 0.12	e

[a]Stimulation by blue light[3,4], post-infrared blue light[2], and pulsed OSL[1].
[b]Signal integration limits 0-0.8 s, background 0.8-1.6 s.
[c]Signal integration limits 0-2 s, background 2-4 s.
[d]Signal integration limits 0-1.6 s, background 36-40 s.
[e]Signal integration limits 0-1.6 s, background 1.6-3.2 s.

Supplementary Table 2. Total organic C (OC) and total N (TN) content, gravimetric OC/TN ratios, $\delta^{13}C$ values of organic carbon, and specific surface area (SSA) of minerals for subsea permafrost drill cores from the Buor Khaya Bay (n.d., not detected; n.a., not analyzed).

Core	Depth m	OC % dry weight	TN % dry weight	OC/TN	$\delta^{13}C$ ‰	SSA $m^2 g^{-1}$ dry weight
4D-13	1.00	0.61	0.08	8.16	-23.47	6.75
4D-13	18.00	2.26	0.19	11.87	-27.49	15.99
4D-13	20.00	0.79	0.10	7.86	-26.95	8.53
4D-13	7.00	0.63	0.08	7.60	-22.96	7.40
4D-13	7.05	0.65	0.08	8.10	-22.81	
4D-13	7.10	0.66	0.09	7.41	-22.73	
4D-13	7.15	0.67	0.09	7.64	-23.30	
4D-13	7.20	0.64	0.08	7.89	-23.34	
4D-13	7.25	0.66	0.08	8.06	-23.08	
4D-13	7.30	0.62	0.08	7.98	-23.48	
4D-13	7.35	0.69	0.09	7.79	-22.95	
4D-13	7.40	0.62	0.08	7.61	-22.80	6.07
4D-13	7.45	0.65	0.08	7.95	-23.13	
4D-13	7.50	0.66	0.08	7.94	-22.77	
4D-13	7.55	0.63	0.08	8.29	-22.71	
4D-13	7.60	0.66	0.09	7.74	-23.23	
4D-13	7.65	0.65	0.08	7.76	-23.01	
4D-13	7.70	0.68	0.08	8.61	-23.45	
4D-13	7.75	0.62	0.08	7.72	-25.88	
4D-13	7.80	0.68	0.10	7.05	-23.00	8.78
4D-13	7.85	0.70	0.08	8.47	-24.90	11.50
4D-13	7.90	0.85	0.07	12.92	-25.38	13.90
4D-13	7.95	0.84	0.07	12.35	-25.33	
4D-13	8.00	0.88	0.07	12.07	-25.26	
4D-13	8.05	0.69	0.06	11.42	-25.08	
4D-13	8.10	0.88	0.08	11.17	-24.80	
4D-13	8.15	0.74	0.07	11.05	-25.82	12.05
4D-13	8.20	0.91	0.08	11.52	-25.88	
4D-13	8.25	0.53	0.04	13.07	-25.11	
4D-13	8.30	0.82	0.07	12.23	-24.91	
4D-13	8.35	0.49	0.05	10.37	-23.92	
4D-13	8.40	0.73	0.05	13.62	-25.62	
4D-13	8.45	0.56	0.05	11.67	-24.13	5.48
4D-13	8.50	0.43	0.04	9.90	-23.41	
4D-13	8.55	0.52	0.06	9.15	-23.23	
4D-13	8.60	0.41	0.04	10.29	-23.27	
4D-13	8.65	0.79	0.07	11.04	-25.59	
4D-13	8.70	0.69	0.06	11.41	-25.19	12.95
4D-13	8.75	0.93	0.08	11.86	-25.19	12.83
4D-13	8.80	0.80	0.07	11.45	-25.17	12.06
4D-13	8.85	1.33	0.10	13.65	-25.72	13.16
4D-13	8.90	0.64	0.05	11.85	-25.29	10.92
4D-13	8.95	0.85	0.07	11.51	-25.10	8.26
4D-13	9.00	0.55	0.05	10.75	-23.61	4.89
4D-13	9.05	0.49	0.05	10.24	-23.37	3.92
4D-13	9.10	0.39	0.04	11.04	-23.56	3.30
4D-13	9.15	0.44	0.04	10.14	-23.56	
4D-13	9.20	0.45	0.04	10.67	-23.28	
4D-13	9.25	0.49	0.04	11.08	-23.38	
4D-13	9.30	0.45	0.04	10.86	-22.88	3.91
4D-13	9.35	0.50	0.05	10.14	-23.61	

4D-13	9.40	0.42	0.04	11.20	-23.28	
4D-13	9.45	0.48	0.05	9.56	-23.73	3.21
4D-13	9.50	0.38	0.04	10.41	-23.31	3.95
4D-13	9.55	2.62	0.24	10.80	-26.66	11.41
4D-13	9.60	0.93	0.10	9.18	-25.01	9.97
4D-13	9.65	0.91	0.10	9.19	-25.15	9.21
4D-13	9.70	1.62	0.16	10.06	-26.71	
4D-13	9.75	0.90	0.11	8.56	-25.01	
4D-13	9.80	0.93	0.10	9.51	-24.90	
4D-13	9.85	0.87	0.11	7.99	-23.57	8.91
4D-13	9.90	1.20	0.12	9.90	-24.94	
4D-13	9.95	0.83	0.10	8.53	-24.39	
4D-13	10.00	0.46	0.05	10.28	-24.92	9.16
2D-13	1.00	1.96	0.17	11.43	-27.07	12.53
2D-13	11.00	0.46	0.03	13.28	-24.07	5.09
2D-13	20.00	0.77	0.10	7.58	-25.50	9.11
2D-13	24.00	1.76	0.13	13.38	-27.58	16.05
2D-13	29.00	0.53	0.08	6.79	-25.10	9.17
2D-13	14.10	0.75	0.05	15.14	-24.96	
2D-13	14.25	0.80	0.05	15.96	-24.35	4.76
2D-13	14.35	0.56	0.04	14.41	-24.35	
2D-13	14.45	0.61	0.04	14.47	-24.69	
2D-13	14.55	0.69	0.05	14.54	-24.52	
2D-13	14.70	0.37	0.04	10.17	-24.23	4.63
2D-13	14.80	0.94	0.05	19.35	-25.27	
2D-13	14.90	1.07	0.07	16.44	-25.97	
2D-13	15.00	0.73	0.06	12.76	-25.00	
2D-13	15.10	0.84	0.07	11.39	-25.40	13.75
2D-13	15.20	0.87	0.09	9.85	-26.11	
2D-13	15.30	0.80	0.08	9.82	-25.92	
2D-13	15.35	0.77	0.08	10.05	-25.59	15.16
2D-13	15.40	0.70	0.06	12.26	-25.12	
2D-13	15.45	0.80	0.06	13.23	-25.49	
2D-13	15.50	0.93	0.06	16.18	-24.94	8.32
2D-13	15.55	0.70	0.06	12.07	-25.21	
2D-13	15.60	0.73	0.05	13.79	-25.08	
2D-13	15.65	0.73	0.05	13.58	-25.19	9.62
2D-13	15.70	0.56	0.07	8.33	-25.38	15.47
2D-13	15.75	0.48	0.06	8.10	-24.99	13.76
2D-13	15.80	0.63	0.07	9.44	-25.68	16.46
2D-13	15.85	0.54	0.06	8.34	-24.93	15.07
2D-13	15.90	0.59	0.06	9.17	-25.12	13.04
2D-13	16.00	0.61	0.06	9.59	-25.35	14.21
2D-13	16.20	0.64	0.06	10.43	-24.84	14.04
2D-13	16.40	0.66	0.06	10.74	-24.90	13.21
2D-13	16.60	0.61	0.06	9.66	-25.32	
2D-13	16.70	0.68	0.06	10.66	-24.96	13.69
2D-13	16.80	0.67	0.06	10.78	-25.07	
2D-13	16.90	0.77	0.07	11.03	-24.70	13.56
4D-12	1.00	1.09	0.12	9.31	-25.49	8.24
4D-12	10.00	0.47	0.05	9.41	-24.81	9.11
4D-12	33.00	2.51	0.22	11.58	-28.34	19.97
4D-12	50.00	0.33	0.03	10.62	-25.02	3.63
4D-12	22.00	0.63	0.10	6.59	-25.58	18.49
4D-12	22.05	0.67	0.10	6.86	-25.76	
4D-12	22.10	0.64	0.10	6.47	-25.64	

4D-12	22.15	0.64	0.10	6.60	-25.42	
4D-12	22.20	0.62	0.10	6.25	-25.39	
4D-12	22.25	0.67	0.10	6.51	-24.84	
4D-12	22.30	0.75	0.10	7.34	-25.47	
4D-12	22.35	0.87	0.11	7.93	-25.49	
4D-12	22.40	0.81	0.11	7.42	-25.75	20.55
4D-12	22.45	0.75	0.11	6.87	-25.58	
4D-12	22.50	0.69	0.10	6.96	-25.39	
4D-12	22.55	0.75	0.11	6.90	-25.46	
4D-12	22.60	0.86	0.11	7.50	-25.38	
4D-12	22.65	0.84	0.12	7.17	-25.48	
4D-12	22.70	0.83	0.11	7.42	-25.72	
4D-12	22.75	0.82	0.11	7.18	-25.67	
4D-12	22.80	0.66	0.10	6.88	-25.71	19.83
4D-12	22.85	0.72	0.10	7.23	-25.87	
4D-12	22.90	0.72	0.10	7.34	-25.53	
4D-12	22.95	0.70	0.10	6.79	-25.68	
4D-12	23.00	0.72	0.10	7.13	-25.64	
4D-12	23.05	0.69	0.10	6.79	-25.50	
4D-12	23.10	0.66	0.09	7.18	-25.51	
4D-12	23.15	0.67	0.09	7.19	-25.29	
4D-12	23.20	0.69	0.10	6.99	-25.47	22.23
4D-12	23.25	0.70	0.09	7.50	-25.60	
4D-12	23.30	0.66	0.09	7.11	-25.60	
4D-12	23.35	0.68	0.09	7.27	-25.74	
4D-12	23.40	0.78	0.10	8.04	-25.83	
4D-12	23.45	0.66	0.10	6.83	-25.50	
4D-12	23.50	0.70	0.10	7.20	-25.39	
4D-12	23.55	0.77	0.10	7.37	-25.34	
4D-12	23.60	0.83	0.10	8.07	-25.41	
4D-12	23.65	0.64	0.09	7.01	-25.44	19.31
4D-12	23.70	0.68	0.09	7.24	-25.48	19.83
4D-12	23.75	0.73	0.10	7.18	-25.02	21.29
4D-12	23.80	0.89	0.10	8.86	-26.25	17.29
4D-12	23.85	0.72	0.10	7.48	-25.43	22.18
4D-12	23.90	0.90	0.11	8.29	-26.48	19.77
4D-12	23.95	0.84	0.10	8.32	-26.12	19.02
4D-12	24.00	0.94	0.10	9.28	-26.82	17.31
4D-12	24.05	0.79	0.11	7.26	-25.80	21.19
4D-12	24.10	0.76	0.10	7.53	-25.52	21.03
4D-12	24.15	0.74	0.10	7.41	-25.15	
4D-12	24.20	0.73	0.10	7.29	-25.43	
4D-12	24.25	0.77	0.10	7.65	-25.34	
4D-12	24.30	0.71	0.09	7.53	-25.69	
4D-12	24.35	0.79	0.10	7.61	-25.72	
4D-12	24.40	0.77	0.10	7.48	-25.54	
4D-12	24.45	0.82	0.11	7.56	-25.88	
4D-12	24.50	0.81	0.11	7.58	-25.42	
4D-12	24.55	0.91	0.12	7.33	-25.39	22.08
4D-12	24.60	0.87	0.12	7.15	-25.70	
4D-12	24.65	0.78	0.11	7.04	-25.66	
4D-12	24.70	0.77	0.11	6.89	-25.30	
4D-12	24.75	0.84	0.11	7.42	-25.37	
4D-12	24.80	0.81	0.11	7.26	-25.27	
4D-12	24.85	0.64	0.10	6.58	-25.31	
4D-12	24.90	0.74	0.10	7.17	-25.41	

4D-12	24.95	0.71	0.10	7.09	-25.45	
4D-12	25.00	0.83	0.10	7.91	-24.97	20.59
1D-14	1.00	4.76	0.34	14.10	-28.08	17.05
1D-14	11.00	3.62	0.16	22.08	-27.35	3.05
1D-14	13.00	0.34	0.03	9.73	-24.59	4.17
1D-14	25.00	0.18	0.02	11.27	-23.71	1.64
1D-14	35.00	0.26	0.01	21.94	-24.31	1.08
5D-13	1.00	2.34	0.21	10.88	-27.79	16.76
5D-13	11.00	0.57	0.04	15.63	-24.59	5.02
5D-13	16.00	0.07	n.d.	n.a.	-24.87	0.67
5D-13	28.00	0.08	n.d.	n.a.	-24.70	0.76
5D-13	40.00	0.14	0.01	15.05	-23.61	1.97

Supplementary Table 3. Correlations between individual parameters measured at the thaw front of the subsea permafrost drill cores 4D-13, 2D-13 and 4D-12 from the Buor-Khaya Bay, including specific surface area of minerals (SSA, $m^2\ g^{-1}$ dry weight), total organic carbon content (OC; % of dry weight), OC/TN ratios, $\delta^{13}C$ values (‰), terrigenous-aquatic *n*-alkane ratios (TAR), $C_{25}/(C_{25}+C_{29})$ *n*-alkane ratios indicative of *Sphagnum* material, S/V and C/V lignin phenol ratios indicative of lignin source vegetation, Sd/Sl, Vd/Vl and 3,5-Bd/V lignin phenol ratios that increase with degradation, CPIs of HMW *n*-alkanes, *n*-alkanoic acids and *n*-alkanols and ratios of HMW *n*-alkanoic acids and *n*-alkanols over *n*-alkanes that decrease with degradation, and sitostanol/*β*-sitosterol ratios at increase with degradation. Presented are Spearman's correlation coefficients for correlations significant at $p < 0.05$ (n.s., not significant).

	SSA	OC	OC/TN	$\delta^{13}C$	TAR	*Sphag.*	S/V	C/V	Sd/Sl	Vd/Vl	3,5-Bd/V	$CPI_{n\text{-alkanes}}$	$CPI_{n\text{-alk. acids}}$	$CPI_{n\text{-alkanols}}$	Alk. ac./ alkanes	Alkanols/ alkanes
OC	+0.337															
OC/TN	−0.580	n.s.														
$\delta^{13}C$	−0.724	−0.501	+0.294													
TAR	+0.598	n.s.	n.s.	−0.704												
Sphagnum	n.s.	n.s.	n.s.	n.s.	n.s.											
S/V	+0.283	+0.299	−0.310	n.s.	n.s.	n.s.										
C/V	+0.547	+0.573	−0.461	−0.558	n.s.	n.s.	+0.568									
Sd/Sl	n.s.	n.s.	n.s.	n.s.	n.s.	n.s.	n.s.	n.s.								
Vd/Vl	n.s.	n.s.	n.s.	n.s.	n.s.	n.s.	n.s.	n.s.	+0.649							
3,5-Bd/V	−0.368	n.s.	n.s.	n.s.	n.s.	n.s.	n.s.	n.s.	n.s.	+0.282						
$CPI_{n\text{-alkanes}}$	n.s.	n.s.	n.s.	n.s.	n.s.	−0.711	n.s.	n.s.	n.s.	n.s.	n.s.					
$CPI_{n\text{-alk. acids}}$	+0.657	n.s.	−0.554	n.s.	n.s.	n.s.	n.s.	n.s.	n.s.	n.s.	n.s.	n.s.				
$CPI_{n\text{-alkanols}}$	n.s.	n.s.	n.s.	n.s.	n.s.	n.s.	n.s.	n.s.	n.s.	n.s.	n.s.	n.s.	n.s.			
Alk. ac./alkanes	n.s.	n.s.	−0.468	n.s.	n.s.	n.s.	n.s.	n.s.	+0.702	+0.656	n.s.	n.s.	+0.547	n.s.		
Alkanols/alkanes	n.s.	n.s.	n.s.	n.s.	n.s.	n.s.	n.s.	n.s.	+0.483	n.s.	n.s.	n.s.	n.s.	n.s.	n.s.	
Sitostanol/-sterol	+0.478	n.s.	−0.435	n.s.	n.s.	+0.577	n.s.	n.s.	n.s.	n.s.	n.s.	−0.612	+0.490	n.s.	n.s.	n.s.

Supplementary Table 4. Statistical analysis of total organic carbon (OC) and total nitrogen (TN) content, OC/TN ratios, $\delta^{13}C$ values of organic carbon and concentrations of lignin, HMW *n*-alkanes, HMW *n*-alkanoic acids and HMW *n*-alkanols, and a range of biomarker proxies, including terrigenous-aquatic ratios (TAR) and $C_{25}/(C_{25}+C_{29})$ *n*-alkane ratios, syringyl/vanillyl (S/V), cinnamyl/vanillyl (C/V), syringic acid/syringaldehyde (Sd/Sl), vanillic acid/vanillin (Vd/Vl), and 3,5-dihydroxybenzoic acid/vanillyl (3,5-Bd/V) lignin phenol ratios, carbon preferences indices (CPI) of HMW *n*-alkanes and HMW *n*-alkanoic acids, ratios of HMW *n*-alkanoic acids and *n*-alkanols over *n*-alkanes, and sitostanol/ *β*-sitosterol, measured in 3 m increments at the thaw front of the subsea permafrost drill cores 4D-13, 2D-13, and 4D-12. "Correlation" columns show Spearman's correlation coefficient and level of significance derived from Spearman's rank sum correlations between individual parameters and depth above the ice-bonded permafrost table (IBPT); positive correlations reflect an increase in the respective parameter with distance above the IBPT. "Difference" columns show the effect direction and level of significance derived from Wilcox tests between core increments above and below the IBPT; positive signs indicate higher values above than below the IBPT. ***, $p < 0.001$; **, $p < 0.01$; *, $p < 0.05$; n.s., not significant.

	4D-13		2D-13		4D-12	
	Correlation	Difference	Correlation	Difference	Correlation	Difference
OC (% dry weight)	n.s.	n.s.	n.s.	n.s.	n.s.	– **
TN (% dry weight)	+0.63 ***	n.s.	–0.53 **	n.s.	n.s.	– *
OC/TN	–0.74 ***	n.s.	+0.67 ***	n.s.	–0.53 ***	– *
$\delta^{13}C$ (‰)	+0.65 ***	n.s.	n.s.	n.s.	n.s.	n.s.
SSA (m^2 g^{-1} dry weight)	n.s.	+ **	n.s.	n.s.	n.s.	n.s.
OC (mg m^{-2} SSA)	n.s.	– **	+0.76 **	n.s.	n.s.	n.s.
Lignin (mg g^{-1} OC)	n.s.	n.s.	–0.74 *	n.s.	n.s.	n.s.
n-Alkanes (mg g^{-1} OC)	n.s.	+ *	n.s.	n.s.	n.s.	n.s.
n-Alkanoic acids (mg g^{-1} OC)	n.s.	n.s.	n.s.	n.s.	n.s.	n.s.
n-Alkanols (mg g^{-1} OC)	n.s.	n.s.	n.s.	n.s.	n.s.	n.s.
TAR	n.s.	n.s.	n.s.	n.s.	n.s.	n.s.
n-Alkanes $C_{25}/(C_{25}+C_{29})$	n.s.	n.s.	n.s.	n.s.	n.s.	n.s.
S/V	n.s.	n.s.	n.s.	n.s.	+0.68 *	n.s.
C/V	n.s.	n.s.	n.s.	n.s.	n.s.	n.s.
Vd/Vl	+0.59 *	n.s.	n.s.	n.s.	n.s.	n.s.
Sd/Sl	n.s.	n.s.	n.s.	n.s.	n.s.	n.s.
3,5-Bd/V	n.s.	n.s.	n.s.	n.s.	n.s.	n.s.
$CPI_{n\text{-alkanes}}$	n.s.	n.s.	n.s.	n.s.	n.s.	n.s.
$CPI_{n\text{-alkanoic acids}}$	n.s.	n.s.	n.s.	n.s.	n.s.	n.s.
n-Alkanoic acids/*n*-alkanes	n.s.	n.s.	n.s.	n.s.	n.s.	n.s.
n-Alkanols/*n*-alkanes	n.s.	n.s.	n.s.	n.s.	n.s.	n.s.
Sitostanol/*β*-sitosterol	n.s.	n.s.	–1.00 *	n.s.	+1.00 *	n.s.

Supplementary Table 5. Production rates of CH_4, CO_2 and N_2O during anoxic incubation of subsea permafrost samples at +4°C (n.d., not detected; n.a, not analyzed).

Sample	Time interval d	CH_4 production nmol g^{-1} OC d^{-1}	CO_2 production µmol g^{-1} OC d^{-1}	N_2O production nmol g^{-1} OC d^{-1}
4D-13 4.55 m	0-1	n.d.	58.2	n.d.
4D-13 4.55 m	1-5	5.7	12.9	2.0
4D-13 4.55 m	5-9	2.0	8.4	-1.9
4D-13 4.55 m	9-16	1.5	2.3	n.d.
4D-13 4.55 m	16-23	1.4	3.3	n.d.
4D-13 4.55 m	23-37	1.3	1.8	n.d.
4D-13 4.55 m	37-114	1.1	0.9	n.d.
4D-13 4.55 m	114-286	0.1	0.6	n.a.
4D-13 4.55 m	377-601	0.1	0.9	n.d.
2D-13 24.70 m	0-1	n.d.	36.3	n.d.
2D-13 24.70 m	1-5	n.d.	4.9	15.9
2D-13 24.70 m	5-9	n.d.	2.8	-11.1
2D-13 24.70 m	9-16	11.3	3.1	-2.2
2D-13 24.70 m	16-23	3.9	1.2	n.d.
2D-13 24.70 m	23-37	3.3	1.6	n.d.
2D-13 24.70 m	37-114	2.6	0.9	n.d.
2D-13 24.70 m	114-286	1.6	0.4	n.a.
2D-13 24.70 m	377-601	0.3	1.5	n.d.
4D-12 27.40 m	0-1	n.d.	51.4	n.d.
4D-12 27.40 m	1-5	n.d.	9.5	3.9
4D-12 27.40 m	5-9	n.d.	5.8	-3.7
4D-12 27.40 m	9-16	4.0	6.8	n.d.
4D-12 27.40 m	16-23	1.6	3.8	n.d.
4D-12 27.40 m	23-37	1.2	2.5	n.d.
4D-12 27.40 m	37-114	0.9	1.1	n.d.
4D-12 27.40 m	114-286	0.8	1.0	n.a.
4D-12 27.40 m	377-601	0.8	0.6	n.d.
1D-14 25.10 m	0-1	n.d.	249.5	n.d.
1D-14 25.10 m	1-5	n.d.	73.1	n.d.
1D-14 25.10 m	5-9	n.d.	41.9	55.4
1D-14 25.10 m	9-16	38.1	38.5	-31.0
1D-14 25.10 m	16-23	12.4	21.4	n.d.
1D-14 25.10 m	23-37	9.8	14.7	n.d.
1D-14 25.10 m	37-114	8.0	10.1	n.d.
1D-14 25.10 m	114-286	3.5	3.2	n.a.
1D-14 25.10 m	377-601	2.2	3.9	n.d.

Supplementary Table 6. Fitted parameters of two-pool models describing CH_4 and CO_2 production over time.

	CH_4			CO_2		
	C_L nmol g^{-1} OC	k_L d^{-1}	k_R nmol g^{-1} OC d^{-1}	C_L µmol g^{-1} OC	k_L d^{-1}	k_R µmol g^{-1} OC d^{-1}
4D-13 4.55 m	158	0.018	0.09	164	0.20	0.81
2D-13 24.70 m	187	0.008	0.35	158	0.13	1.20
4D-12 27.40 m	315	0.018	1.04	105	0.10	0.53
1D-14 25.10 m	923	0.025	2.63	1544	0.05	3.82

Supplementary Table 7. Organic carbon concentration and isotopic composition of CH_4 produced by organic matter decomposition in thawed subsea permafrost during 335 days of incubation.

Sample	OC<br>%	δD VSMOW<br>‰	$\delta^{13}C$ VPDB<br>‰
4D-13 4.55 m	2.26	-317	-65.05
2D-13 24.70 m	0.22	-289	-61.45
4D-12 27.40 m	0.91	-304	-61.45
1D-14 25.10 m	0.07	-289	-72.31

Supplementary Table 8. Grain size distribution of subsea permafrost drill cores from the Buor Khaya Bay (original data on 4D-13, 2D-13, 4D-12 and 1D-14 from refs. [12,13]). Peaks of grain size distribution were determined using Gradistat v8[14].

Core	Depth<br>m	> 10 mm<br>%	5-10 mm<br>%	2-5 mm<br>%	1-2 mm<br>%	0.5-1 mm<br>%	250-500 µm<br>%	100-250 µm<br>%	50-100 µm<br>%	10-50 µm<br>%	5-10 µm<br>%	1-5 µm<br>%	< 1 µm<br>%	Grain size distribution peaks
4D-13	0.20-0.40	0.00	0.00	0.00	0.00	10.58	31.54	16.29	3.56	15.27	7.94	12.15	2.67	375 µm, 7.5 µm
4D-13	7.00-7.20	0.00	0.00	0.00	0.00	2.76	10.80	15.64	15.30	34.09	7.39	11.18	2.84	75 µm
4D-13	8.00-8.20	0.00	0.00	0.00	0.00	3.31	13.32	15.48	12.28	32.67	7.51	12.32	3.11	30 µm, 375 µm
4D-13	9.20-9.40	0.00	0.00	0.00	0.00	11.65	29.44	13.72	7.74	21.49	4.99	8.70	2.27	375 µm, 30 µm
4D-13	9.80-10.00	0.00	0.00	0.00	0.00	3.95	21.81	20.82	11.68	23.52	6.44	9.29	2.49	375 µm
4D-13	11.20-11.40	0.00	0.00	0.00	0.00	9.76	27.90	17.24	3.78	14.99	8.03	14.26	4.04	375 µm, 7.5 µm
4D-13	13.60-13.80	0.00	0.00	0.00	0.00	2.46	21.16	37.73	11.80	13.02	4.35	7.67	1.81	175 µm
4D-13	15.20-15.40	0.00	0.00	0.00	0.00	13.43	35.60	13.59	7.92	16.40	4.21	7.17	1.68	375 µm
4D-13	16.20-16.40	0.00	0.00	0.00	0.00	9.14	31.14	21.46	5.93	11.96	5.86	11.18	3.33	375 µm, 7.5 µm
4D-13	17.00-17.20	0.00	0.00	0.00	0.00	5.28	27.39	23.38	8.65	14.06	6.39	11.60	3.25	375 µm, 7.5 µm
4D-13	17.40-17.60	0.00	0.00	0.00	0.00	8.08	24.11	17.57	6.65	18.76	7.47	13.30	4.06	375 µm, 30 µm
4D-13	18.00-18.20	0.00	0.00	0.00	0.00	1.33	5.81	6.09	8.46	44.91	11.99	17.28	4.13	30 µm, 375 µm
4D-13	18.30-18.50	0.00	0.00	0.00	0.00	0.00	0.00	0.84	4.53	47.70	15.64	24.84	6.45	30 µm
4D-13	18.60-19.00	0.00	0.00	0.00	0.00	0.29	0.83	2.30	4.62	49.99	16.52	21.04	4.41	30 µm
4D-13	20.40-20.60	0.00	0.00	0.00	0.00	11.94	34.01	13.24	2.62	10.76	7.65	15.22	4.56	375 µm, 7.5 µm
2D-13	0.30-0.70	0.00	0.00	0.00	0.00	0.78	8.94	60.01	18.12	6.10	1.57	3.35	1.13	175 µm
2D-13	1.30-1.40	0.00	0.00	0.00	0.00	0.48	9.56	61.56	17.11	5.38	1.54	3.30	1.07	175 µm
2D-13	1.50-1.60	0.00	0.00	0.00	0.00	0.76	7.12	55.38	25.59	5.01	1.49	3.49	1.16	175 µm
2D-13	7.20-7.30	0.00	0.00	0.00	0.00	0.00	0.00	0.00	0.12	36.92	21.42	33.94	7.60	7.5 µm
2D-13	7.60-7.70	0.00	0.00	0.00	0.00	0.00	0.00	0.00	0.09	36.91	20.95	33.60	8.45	7.5 µm
2D-13	8.00-8.20	0.00	0.00	0.00	0.00	0.00	0.00	0.00	0.18	40.58	19.52	31.48	8.24	7.5 µm
2D-13	8.40-8.60	0.00	0.00	0.00	0.00	0.00	0.00	0.00	0.00	21.08	27.11	40.90	10.91	7.5 µm
2D-13	8.70-8.80	0.00	0.00	0.00	0.00	0.00	0.00	0.00	0.13	37.43	20.48	33.19	8.77	7.5 µm
2D-13	9.50-9.60	0.00	0.00	0.00	0.00	0.00	0.00	0.00	0.05	31.99	21.12	37.37	9.47	7.5 µm
2D-13	11.00-11.20	0.00	0.00	0.00	0.00	0.00	0.00	0.00	0.00	30.49	21.21	38.16	10.14	7.5 µm
2D-13	12.30-12.50	0.00	0.00	0.00	0.00	0.00	0.00	0.00	0.05	33.57	21.28	36.14	8.96	7.5 µm
2D-13	13.00-13.20	0.00	0.00	0.00	0.00	0.00	0.00	0.00	0.13	33.65	21.04	36.28	8.90	7.5 µm
2D-13	13.90-14.10	0.00	0.00	0.00	0.00	0.00	0.00	0.00	0.00	35.09	20.93	35.03	8.95	7.5 µm
2D-13	15.10-15.30	0.00	0.00	0.00	0.00	0.00	0.00	0.00	0.14	35.54	21.39	35.21	7.72	7.5 µm
2D-13	15.40-15.50	0.00	0.00	0.00	0.00	8.75	29.50	26.66	12.62	11.97	2.86	5.80	1.84	375 µm
2D-13	15.70-15.80	0.00	0.00	0.00	0.00	9.05	27.90	22.20	12.16	13.49	4.23	8.45	2.52	375 µm
2D-13	15.90-16.00	0.00	0.00	0.00	0.00	5.85	23.40	26.41	17.38	14.84	3.07	6.91	2.14	375 µm
2D-13	16.80-17.00	0.00	0.00	0.00	0.00	3.98	15.81	26.50	17.89	17.81	5.48	9.78	2.75	175 µm
2D-13	17.60-17.80	0.00	0.00	0.00	0.00	16.09	41.88	15.00	5.64	7.99	3.66	7.58	2.16	375 µm
2D-13	18.00-18.20	0.00	0.00	0.00	0.00	0.00	5.87	17.46	15.91	33.81	10.53	13.42	3.00	75 µm

2D-13	18.40-18.60	0.00	0.00	0.00	0.00	0.19	1.75	9.01	13.51	42.55	12.42	15.58	4.99	30 µm
2D-13	19.20-19.40	0.00	0.00	0.00	0.00	7.90	18.87	15.53	12.98	26.87	6.15	9.09	2.61	375 µm, 75 µm
2D-13	20.00-20.20	0.00	0.00	0.00	0.00	0.00	12.60	32.24	18.32	20.21	5.38	8.82	2.43	175 µm
2D-13	20.30-20.50	0.00	0.00	0.00	0.00	1.10	2.29	5.39	9.43	47.56	13.56	15.92	4.75	30 µm
2D-13	20.80-21.00	0.00	0.00	0.00	0.00	1.13	6.50	3.23	5.04	50.95	10.59	17.67	4.89	30 µm, 375 µm
2D-13	21.80-22.00	0.00	0.00	0.00	0.00	0.00	0.00	0.60	6.79	58.97	10.61	18.04	4.99	30 µm
2D-13	22.20-22.40	0.00	0.00	0.00	0.00	1.18	4.67	1.95	5.44	52.55	10.38	18.40	5.43	30 µm, 375 µm
2D-13	22.70-22.90	0.00	0.00	0.00	0.00	3.64	8.93	1.57	3.60	51.13	9.49	16.79	4.85	30 µm, 375 µm
2D-13	23.00-23.20	0.00	0.00	0.00	0.00	1.58	5.22	1.48	5.01	54.17	9.66	17.65	5.23	30 µm, 375 µm
2D-13	24.00-24.20	0.00	0.00	0.00	0.00	0.00	0.00	0.00	1.91	69.04	9.44	15.53	4.08	30 µm
2D-13	24.50-24.70	0.00	0.00	0.00	0.00	0.00	0.00	0.00	2.13	68.99	9.13	15.18	4.57	30 µm
2D-13	25.00-25.30	0.00	0.00	0.00	0.00	0.00	0.00	0.00	2.64	69.99	8.49	14.59	4.29	30 µm
2D-13	25.50-25.70	0.00	0.00	0.00	0.00	0.00	0.00	0.01	3.89	72.03	6.50	13.49	4.08	30 µm
2D-13	28.30-28.50	0.00	0.00	0.00	0.00	7.57	20.32	28.88	21.87	10.46	2.72	6.27	1.91	75 µm
2D-13	29.00-29.00	0.00	0.00	0.00	0.00	5.19	16.63	32.30	24.46	9.11	2.64	7.31	2.36	75 µm
2D-13	29.40-29.60	0.00	0.00	0.00	0.00	0.00	0.00	0.11	4.42	59.48	10.67	19.62	5.70	30 µm
2D-13	29.60-29.80	0.00	0.00	0.00	0.00	0.00	0.00	0.40	4.26	59.49	11.02	19.45	5.38	30 µm
2D-13	30.00-30.20	0.00	0.00	0.00	0.00	0.00	0.00	0.00	4.27	60.11	10.61	19.43	5.58	30 µm
4D-12	0.00-0.40	0.00	0.00	0.00	0.00	0.00	0.00	0.00	0.00	2.52	27.11	59.38	10.99	7.5 µm
4D-12	1.00-1.10	0.00	0.00	0.00	0.00	0.00	0.00	0.00	0.00	35.76	24.78	33.72	5.74	7.5 µm
4D-12	3.00-3.05	0.00	0.00	0.00	0.00	0.00	0.00	0.00	0.00	23.01	28.63	41.40	6.96	7.5 µm
4D-12	3.45-3.50	0.00	0.00	0.00	0.00	0.00	0.00	0.00	0.00	26.80	26.39	39.38	7.43	7.5 µm
4D-12	3.65-3.70	0.00	0.00	0.00	0.00	0.00	0.00	0.00	0.00	5.30	30.30	54.23	10.17	7.5 µm
4D-12	4.00-4.10	0.00	0.00	0.00	0.00	0.00	0.00	0.00	3.52	47.81	15.94	25.61	7.12	30 µm
4D-12	4.15-4.20	0.00	0.00	0.00	0.00	0.00	0.00	0.00	0.00	31.23	26.38	35.93	6.46	7.5 µm
4D-12	5.20-5.25	0.00	0.00	0.00	0.00	0.00	0.00	0.00	1.46	59.18	14.29	19.78	5.29	30 µm
4D-12	6.00-6.05	0.00	0.00	0.00	0.00	0.00	0.00	0.00	2.53	55.66	15.09	20.31	6.41	30 µm
4D-12	6.45-6.50	0.00	0.00	0.00	0.00	0.00	0.00	0.00	0.00	33.81	23.10	33.94	9.15	7.5 µm
4D-12	6.90-6.95	0.00	0.00	0.00	0.00	0.00	0.00	0.00	0.00	34.55	20.75	35.39	9.31	7.5 µm
4D-12	7.30-7.35	0.00	0.00	1.56	2.87	4.55	14.54	24.36	3.11	6.27	15.36	22.55	4.84	175 µm, 7.5 µm
4D-12	12.00-12.05	0.00	0.00	0.00	0.00	0.00	0.00	0.00	0.00	15.97	23.90	48.46	11.67	7.5 µm
4D-12	14.00-14.05	0.00	0.00	0.00	0.00	0.00	0.00	0.00	0.00	9.11	31.25	50.68	8.96	7.5 µm
4D-12	16.00-16.10	0.00	0.00	1.68	8.52	24.77	33.32	28.05	2.54	1.12	0.00	0.00	0.00	375 µm
4D-12	16.40-16.50	0.00	0.00	0.14	0.77	1.93	6.65	82.85	7.27	0.39	0.00	0.00	0.00	175 µm
4D-12	19.00-19.10	0.00	0.00	0.00	0.00	0.00	0.00	0.00	0.00	11.11	23.98	51.54	13.37	7.5 µm
4D-12	19.50-19.60	0.00	0.00	0.00	0.00	0.00	0.00	0.00	0.00	14.78	24.13	49.23	11.86	7.5 µm
4D-12	20.40-20.45	0.00	0.00	0.00	0.00	0.00	0.00	0.05	13.02	48.25	8.96	22.40	7.32	30 µm, 3 µm
4D-12	20.55-20.60	0.00	0.00	2.26	17.13	23.90	34.80	16.73	4.25	0.93	0.00	0.00	0.00	375 µm
4D-12	20.90-20.95	0.00	0.00	0.00	12.20	12.26	10.66	52.23	11.95	0.70	0.00	0.00	0.00	175 µm, 750 µm
4D-12	22.40-22.45	0.00	0.00	1.95	10.77	8.15	11.54	44.92	21.54	1.13	0.00	0.00	0.00	175 µm, 1.5 mm

4D-12	23.45-23.50	0.00	0.00	0.00	0.00	0.00	0.00	0.00	0.00	20.70	25.68	44.50	9.12	7.5 µm
4D-12	24.05-24.10	0.00	0.00	0.00	0.00	0.00	0.00	0.00	0.00	44.47	19.51	28.76	7.26	7.5 µm
4D-12	24.50-24.55	0.00	0.00	0.00	0.00	0.00	0.00	0.00	2.92	53.57	14.96	23.10	5.45	30 µm
4D-12	25.00-25.10	0.00	0.00	0.00	0.00	0.00	0.00	0.00	0.00	19.96	25.52	41.10	13.42	7.5 µm
4D-12	26.00-26.10	0.00	0.00	0.00	0.00	0.00	0.00	0.00	0.00	41.74	20.74	30.34	7.18	7.5 µm
4D-12	26.10-26.15	0.00	0.00	0.00	0.00	0.00	0.00	0.00	2.20	62.36	12.11	18.37	4.96	30 µm
4D-12	26.20-26.30	0.00	0.00	0.00	0.00	0.00	0.00	0.00	0.20	43.41	18.23	30.12	8.04	30 µm
4D-12	27.10-27.20	0.00	0.00	4.06	12.88	14.42	14.69	44.06	8.31	1.58	0.00	0.00	0.00	175 µm
4D-12	28.80-28.90	0.00	0.00	2.07	8.11	11.18	9.90	38.47	0.33	4.49	7.70	14.04	3.71	175 µm, 750 µm, 7.5 µm
4D-12	29.00-29.10	0.00	0.00	1.93	6.79	15.88	12.11	16.60	0.35	23.11	9.39	11.37	2.47	750 µm, 175 µm, 30 µm
4D-12	30.00-30.10	0.00	0.00	0.00	0.00	0.00	0.00	0.00	0.17	47.92	18.45	27.31	6.15	30 µm
4D-12	31.00-31.05	0.00	0.00	0.00	0.00	0.00	0.00	0.00	0.00	10.93	23.02	49.95	16.10	7.5 µm
4D-12	31.40-31.45	0.00	0.00	0.00	0.00	0.00	0.00	0.00	0.00	27.79	28.83	35.48	7.90	7.5 µm
4D-12	31.50-31.60	0.00	0.00	0.00	0.00	0.00	0.00	0.00	0.32	63.09	12.94	18.17	5.48	30 µm
4D-12	32.10-32.15	0.00	0.00	0.00	0.00	0.24	2.04	5.50	4.54	41.33	16.96	22.94	6.45	30 µm
4D-12	32.20-32.25	0.00	0.00	0.00	0.00	0.00	0.00	0.00	0.18	46.72	18.28	28.26	6.56	30 µm
4D-12	33.50-33.55	0.00	0.00	3.30	8.25	6.97	15.14	20.26	5.10	10.56	10.22	16.16	4.04	175 µm, 7.5 µm, 1.5 mm
4D-12	33.58-33.62	0.00	0.00	0.00	0.00	0.00	0.00	0.00	0.00	30.69	25.89	36.96	6.46	7.5 µm
4D-12	36.50-36.60	0.00	0.00	9.39	14.09	10.06	27.55	26.75	0.40	1.32	3.14	5.99	1.32	375 µm, 1.5 mm
4D-12	38.50-38.60	0.00	0.00	0.43	1.90	5.90	27.66	59.11	4.50	0.50	0.00	0.00	0.00	175 µm
4D-12	39.50-39.60	0.00	0.00	0.06	1.46	5.88	17.78	68.10	6.38	0.34	0.00	0.00	0.00	175 µm
4D-12	40.50-40.60	0.00	0.00	0.00	0.16	4.61	56.50	36.46	1.99	0.28	0.00	0.00	0.00	375 µm
4D-12	41.00-41.10	0.00	0.00	0.06	0.39	5.53	30.88	55.26	6.91	0.97	0.00	0.00	0.00	175 µm
4D-12	41.80-41.90	0.00	0.00	0.00	0.37	3.22	23.56	52.36	0.67	5.16	4.52	8.21	1.93	175 µm
4D-12	43.10-43.20	0.00	0.00	0.00	0.21	3.46	30.69	63.59	1.90	0.15	0.00	0.00	0.00	175 µm
4D-12	44.10-44.15	0.00	0.00	0.00	0.29	0.77	17.72	77.85	3.06	0.31	0.00	0.00	0.00	175 µm
4D-12	44.60-44.65	0.00	0.00	0.00	0.24	1.49	25.55	69.68	2.77	0.27	0.00	0.00	0.00	175 µm
4D-12	45.00-45.10	0.00	0.00	0.96	3.56	6.36	3.97	80.05	4.93	0.17	0.00	0.00	0.00	175 µm
4D-12	46.50-46.55	0.00	0.00	0.00	0.00	1.38	19.22	76.37	2.76	0.28	0.00	0.00	0.00	175 µm
4D-12	47.00-47.10	0.00	0.00	0.00	0.31	2.43	4.07	84.68	0.45	2.73	1.68	2.98	0.67	175 µm
4D-12	47.50-47.60	0.00	0.00	0.00	0.21	1.52	21.38	69.65	6.62	0.62	0.00	0.00	0.00	175 µm
4D-12	48.00-48.10	0.00	0.00	0.00	0.48	2.48	9.00	68.00	18.68	1.37	0.00	0.00	0.00	175 µm
4D-12	49.00-49.10	0.00	0.00	0.00	0.28	0.65	2.42	85.48	10.60	0.57	0.00	0.00	0.00	175 µm
4D-12	49.50-49.55	0.00	0.00	0.00	0.22	0.31	0.44	67.80	29.71	1.53	0.00	0.00	0.00	175 µm
4D-12	49.60-49.70	0.00	0.00	0.00	0.52	3.81	27.05	59.39	8.53	0.70	0.00	0.00	0.00	175 µm
4D-12	50.00-50.10	0.00	0.00	0.29	0.24	0.29	1.83	43.77	1.00	1.30	10.37	31.61	9.30	175 µm, 3 µm
4D-12	51.00-51.10	0.00	0.00	0.00	0.00	0.00	0.00	0.00	0.00	17.91	22.83	47.16	12.10	7.5 µm
4D-12	54.00-54.10	0.00	0.00	0.00	0.14	0.35	1.04	71.87	25.70	0.90	0.00	0.00	0.00	175 µm

Core	Depth<br>m	> 1 mm %	0.5-1 mm %	250-500 µm %	100-250 µm %	50-100 µm %	10-50 µm %	5-10 µm %	1-5 µm %	< 1 µm %	Grain size distribution peaks
1D-14	0.00	0.13	14.71	3.78	33.68	19.55	25.64	2.51	0.13	14.71	7.5 µm, 550 µm
1D-14	0.55	1.31	84.92	1.50	6.07	2.33	3.41	0.46	1.31	84.92	550 µm
1D-14	0.96	0.45	28.01	11.74	38.34	7.81	11.57	2.08	0.45	28.01	30 µm
1D-14	1.90	1.31	82.73	2.01	7.90	2.19	3.22	0.64	1.31	82.73	550 µm
1D-14	2.96	1.02	62.60	3.46	18.23	6.07	7.54	1.08	1.02	62.60	550 µm, 30 µm
1D-14	4.15	4.60	32.18	4.42	32.51	9.81	14.08	2.40	4.60	32.18	30 µm, 550 µm
1D-14	4.48	0.65	42.07	2.74	27.23	9.66	15.70	1.95	0.65	42.07	550 µm, 30 µm
1D-14	4.96	2.12	71.98	1.79	12.31	4.50	6.40	0.90	2.12	71.98	550 µm, 30 µm
1D-14	5.46	5.41	46.50	2.93	22.50	8.19	12.40	2.07	5.41	46.50	550 µm, 30 µm
1D-14	8.78	5.17	59.86	9.35	12.14	4.13	7.68	1.67	5.17	59.86	550 µm
1D-14	10.39	0.21	62.28	8.05	18.27	4.55	5.65	0.99	0.21	62.28	550 µm
1D-14	11.42	2.51	79.94	2.35	8.18	3.29	3.26	0.47	2.51	79.94	550 µm
1D-14	12.00	18.95	44.16	6.19	17.26	5.10	7.10	1.24	18.95	44.16	1500 µm, 30 µm
1D-14	12.18	0.32	45.41	10.95	24.41	7.44	9.91	1.56	0.32	45.41	550 µm
1D-14	13.58	2.12	62.30	4.05	12.23	7.90	10.20	1.20	2.12	62.30	550 µm, 7.5 µm
1D-14	13.91	24.10	70.61	0.57	1.88	1.03	1.57	0.24	24.10	70.61	1500 µm
1D-14	14.71	2.17	80.42	4.61	6.52	2.08	3.48	0.72	2.17	80.42	550 µm
1D-14	14.86	0.27	31.10	6.43	37.53	10.89	11.61	2.17	0.27	31.10	30 µm, 550 µm
1D-14	16.31	0.24	19.35	10.81	47.23	10.07	10.22	2.08	0.24	19.35	30 µm
1D-14	17.06	0.70	64.75	8.19	13.43	5.03	6.71	1.19	0.70	64.75	550 µm
1D-14	17.43	0.53	59.89	5.28	19.09	6.04	7.90	1.27	0.53	59.89	550 µm, 30 µm
1D-14	17.96	5.88	67.49	3.56	13.03	3.67	5.39	0.98	5.88	67.49	550 µm, 30 µm
1D-14	19.01	4.25	89.21	1.10	3.02	1.11	1.16	0.15	4.25	89.21	550 µm
1D-14	19.14	22.70	68.81	1.66	3.47	1.46	1.68	0.22	22.70	68.81	1500 µm
1D-14	20.59	0.11	16.24	21.51	38.53	9.51	12.04	2.06	0.11	16.24	75 µm
1D-14	20.87	2.49	66.32	4.70	14.33	4.31	6.51	1.34	2.49	66.32	550 µm, 30 µm
1D-14	21.19	3.01	50.54	8.20	21.63	7.18	8.44	1.00	3.01	50.54	550 µm, 30 µm
1D-14	23.66	0.01	88.50	5.18	3.58	1.09	1.40	0.24	0.01	88.50	550 µm
1D-14	24.60	0.65	81.38	1.77	7.56	3.20	4.67	0.77	0.65	81.38	550 µm
1D-14	27.01	0.02	99.20	0.15	0.36	0.11	0.13	0.03	0.02	99.20	550 µm
1D-14	31.54	15.44	58.52	3.00	13.66	3.95	4.55	0.88	15.44	58.52	550 µm, 30 µm
1D-14	33.13	0.29	96.13	0.21	1.66	0.75	0.81	0.15	0.29	96.13	550 µm
1D-14	34.47	7.40	90.01	0.65	1.21	0.29	0.37	0.07	7.40	90.01	550 µm
1D-14	35.97	0.39	66.36	8.45	15.37	4.06	4.50	0.87	0.39	66.36	550 µm
1D-14	37.45	0.06	92.38	1.47	3.33	1.06	1.43	0.27	0.06	92.38	550 µm
1D-14	37.82	0.01	28.69	10.10	33.72	12.46	13.13	1.89	0.01	28.69	30 µm

5D-13	2.40	0.60	5.35	4.99	44.18	17.58	23.77	3.53	0.60	5.35	30 µm
5D-13	2.95	0.50	13.75	6.11	41.82	15.21	19.79	2.82	0.50	13.75	30 µm
5D-13	3.50	1.61	6.88	3.02	38.39	20.95	26.32	2.83	1.61	6.88	7.5 µm
5D-13	4.10	0.00	4.86	4.37	44.55	19.11	24.27	2.84	0.00	4.86	30 µm
5D-13	5.30	20.04	19.75	4.05	28.45	10.26	14.96	2.49	20.04	19.75	1500 µm, 30 µm
5D-13	9.65	0.55	46.00	20.80	16.48	5.21	9.25	1.71	0.55	46.00	75 µm
5D-13	10.10	0.35	16.10	26.78	27.87	10.01	16.08	2.81	0.35	16.10	75 µm
5D-13	11.00	0.98	32.50	24.87	24.21	6.11	9.52	1.81	0.98	32.50	75 µm
5D-13	11.90	5.10	6.62	13.21	45.78	11.53	15.09	2.67	5.10	6.62	30 µm, 1500 µm
5D-13	12.85	45.53	37.07	3.33	7.15	2.31	3.53	0.75	45.53	37.07	1500 µm
5D-13	13.70	10.53	72.31	6.91	5.63	1.60	2.54	0.48	10.53	72.31	550 µm
5D-13	19.55	4.68	92.08	1.34	1.02	0.31	0.49	0.08	4.68	92.08	550 µm
5D-13	25.10	6.58	80.10	4.81	4.39	1.51	2.29	0.32	6.58	80.10	550 µm
5D-13	25.85	10.51	88.09	0.22	0.61	0.20	0.31	0.06	10.51	88.09	550 µm

Supplementary Table 9. Concentrations of vanillin (Vl), acetovanillone (Vn), vanillic acid (Vd), syringaldehyde (Sl), acetosyringone (Sn), syringic acid (Sd), *p*-coumaric acid (*p*Cd), ferulic acid (Fd), benzoic acid (Bd), *m*-hydroxybenzoic acid (*m*-Bd), 3,5-dihydroxybenzoic acid (3,5-Bd), *p*-hydroxybenzaldehyde (Pl), *p*-hydroxyacetophenone (Pn), and *p*-hydroxybenzoic acid (Pd) for 3 m increments at the thaw front of the subsea permafrost drill cores 4D-13, 2D-13, 4D-12 from the Buor Khaya Bay, as well as in the incubated samples.

Core	Depth	Vl	Vn	Vd	Sl	Sn	Sd	*p*Cd	Fd	Bd	*m*-Bd	3,5-Bd	Pl	Pn	Pd
	m							mg g^{-1} OC							
4D-13	7.00	0.21	0.05	0.22	0.10	0.02	0.06	0.02	0.02	0.11	0.04	0.17	0.16	0.04	0.28
4D-13	7.40	0.13	0.05	0.15	0.08	0.04	0.07	0.04	0.04	0.10	0.07	0.05	0.17	0.07	0.22
4D-13	7.80	3.65	0.84	1.47	3.20	0.84	0.89	0.78	1.66	0.38	0.19	0.54	1.37	0.29	2.49
4D-13	7.85	5.05	1.22	2.30	3.95	1.03	1.22	0.78	1.51	0.64	0.23	0.45	1.55	0.32	3.15
4D-13	7.90	2.14	0.46	0.95	1.48	0.39	0.47	0.50	0.86	0.27	0.13	0.14	0.73	0.13	2.09
4D-13	8.15	4.04	0.88	1.44	2.74	0.67	0.69	0.43	0.72	0.48	0.24	0.56	1.26	0.29	2.58
4D-13	8.45	3.17	0.58	1.14	1.53	0.34	0.38	0.14	0.24	0.61	0.10	0.26	0.71	0.15	1.02
4D-13	8.70	2.30	0.40	0.98	1.43	0.31	0.46	0.37	0.66	0.25	0.11	0.13	0.77	0.13	2.34
4D-13	8.75	4.64	1.13	1.82	3.34	0.86	0.94	0.54	1.07	0.43	0.20	0.55	1.19	0.26	2.44
4D-13	8.80	4.91	1.17	1.91	3.36	0.89	0.90	0.52	0.98	0.42	0.22	0.48	1.31	0.29	2.84
4D-13	8.85	1.63	0.36	0.72	0.95	0.26	0.33	0.35	0.58	0.30	0.11	0.13	0.60	0.13	2.10
4D-13	8.90	8.05	1.93	2.93	5.40	1.40	1.38	0.78	1.71	0.60	0.31	0.94	2.07	0.49	4.36
4D-13	8.95	2.99	0.58	1.21	1.97	0.57	0.69	0.43	0.74	0.41	0.16	0.38	1.00	0.18	1.68
4D-13	9.00	0.63	0.17	0.36	0.26	0.10	0.18	0.12	0.14	0.19	0.14	0.11	0.42	0.14	0.68
4D-13	9.05	0.43	0.09	0.23	0.19	0.04	0.08	0.03	0.04	0.06	0.05	0.14	0.16	0.04	0.28
4D-13	9.10	0.24	0.12	0.20	0.14	0.09	0.12	0.08	0.09	0.19	0.16	0.18	0.25	0.13	0.27
4D-13	9.30	0.17	0.03	0.10	0.08	0.01	0.03	0.02	0.03	0.04	0.02	0.00	0.15	0.02	0.24
4D-13	9.45	0.15	0.03	0.08	0.09	0.03	0.03	0.01	0.02	0.27	0.03	0.09	0.06	0.03	0.08
4D-13	9.50	0.63	0.13	0.30	0.32	0.07	0.10	0.05	0.07	0.14	0.07	0.16	0.32	0.08	0.47
4D-13	9.55	1.42	0.33	0.83	1.35	0.48	0.64	1.21	2.82	0.12	0.09	0.15	1.47	0.17	3.10
4D-13	9.60	3.87	0.94	1.60	4.85	1.30	1.31	1.29	4.42	0.38	0.18	0.56	2.47	0.49	3.71
4D-13	9.65	4.00	0.99	1.36	4.05	1.08	0.99	0.85	2.53	0.40	0.17	0.59	1.98	0.41	2.90
4D-13	9.85	0.71	0.11	0.31	0.50	0.09	0.19	0.24	0.49	0.06	0.04	0.04	0.48	0.06	0.90
4D-13	10.00	3.62	0.84	1.75	2.50	0.62	0.91	0.56	0.93	0.57	0.17	0.42	1.51	0.26	2.65
2D-13	14.25	0.67	0.10	0.24	0.30	0.04	0.06	0.06	0.09	0.07	0.03	0.05	0.47	0.07	0.63
2D-13	14.70	0.55	0.05	0.20	0.22	0.01	0.07	0.03	0.04	0.02	0.03	0.02	0.35	0.05	0.51
2D-13	15.10	0.62	0.13	0.32	0.42	0.09	0.10	0.11	0.12	0.15	0.05	0.08	0.37	0.07	0.73
2D-13	15.35	0.71	0.14	0.31	0.45	0.09	0.10	0.11	0.10	0.12	0.05	0.07	0.31	0.07	0.75
2D-13	15.50	0.43	0.08	0.22	0.18	0.03	0.04	0.06	0.10	0.08	0.04	0.07	0.18	0.04	0.43
2D-13	15.65	0.73	0.10	0.42	0.27	0.05	0.07	0.08	0.11	0.02	0.04	0.07	0.24	0.04	1.20
2D-13	15.70	1.15	0.20	0.48	0.70	0.17	0.17	0.18	0.21	0.29	0.05	0.07	0.42	0.07	0.71
2D-13	15.75	0.87	0.10	0.34	0.41	0.06	0.12	0.10	0.11	0.09	0.03	0.02	0.44	0.05	0.44
2D-13	15.80	0.91	0.18	0.36	0.58	0.16	0.15	0.17	0.28	0.15	0.05	0.07	0.38	0.06	0.45
2D-13	15.85	0.92	0.14	0.40	0.52	0.09	0.15	0.12	0.13	0.07	0.05	0.06	0.41	0.06	0.48
2D-13	15.90	0.92	0.19	0.40	0.56	0.13	0.15	0.16	0.21	0.13	0.04	0.07	0.35	0.06	0.40
2D-13	16.00	0.89	0.15	0.37	0.50	0.13	0.13	0.15	0.23	0.09	0.04	0.07	0.27	0.06	0.62
2D-13	16.20	0.95	0.18	0.40	0.60	0.15	0.15	0.18	0.23	0.17	0.05	0.08	0.27	0.05	0.80
2D-13	16.40	1.10	0.19	0.44	0.67	0.15	0.16	0.18	0.32	0.08	0.06	0.11	0.35	0.07	0.81
2D-13	16.70	1.12	0.22	0.41	0.56	0.13	0.14	0.11	0.22	0.16	0.05	0.07	0.31	0.07	0.48
2D-13	16.90	0.59	0.10	0.24	0.31	0.06	0.08	0.09	0.11	0.02	0.04	0.06	0.28	0.04	0.34
4D-12	22.00	0.82	0.27	0.46	0.57	0.22	0.24	0.24	0.42	0.14	0.14	0.15	0.43	0.14	0.50
4D-12	22.40	1.29	0.25	0.55	1.24	0.38	0.30	0.42	0.97	0.05	0.08	0.12	0.67	0.12	0.58
4D-12	22.80	1.01	0.26	0.52	0.75	0.28	0.29	0.30	0.64	0.11	0.07	0.09	0.47	0.10	0.61
4D-12	23.20	1.52	0.37	0.71	1.18	0.34	0.33	0.23	0.65	0.27	0.23	0.18	0.56	0.12	0.98
4D-12	23.65	0.86	0.21	0.38	0.51	0.14	0.14	0.16	0.27	0.19	0.33	0.05	0.39	0.07	0.87
4D-12	23.70	1.00	0.23	0.50	0.67	0.20	0.20	0.22	0.51	0.20	0.50	0.15	0.43	0.10	1.41
4D-12	23.75	0.88	0.16	0.40	0.68	0.16	0.16	0.19	0.33	0.25	0.26	0.04	0.46	0.07	0.77
4D-12	23.80	2.36	0.57	1.12	0.95	0.25	0.26	0.36	2.43	0.16	0.32	0.26	0.49	0.17	0.87

4D-12	23.85	0.98	0.18	0.41	0.71	0.16	0.17	0.18	0.39	0.14	0.12	0.07	0.50	0.07	0.52
4D-12	23.90	0.79	0.25	0.49	0.34	0.18	0.22	0.26	0.90	0.20	0.19	0.16	0.34	0.15	0.58
4D-12	23.95	2.21	0.55	1.08	0.43	0.13	0.16	0.22	0.94	0.14	0.11	0.13	0.36	0.10	0.60
4D-12	24.00	1.16	0.23	0.72	0.25	0.06	0.10	0.20	1.41	0.04	0.08	0.06	0.23	0.06	0.57
4D-12	24.05	1.08	0.20	0.49	0.85	0.21	0.23	0.23	0.57	0.07	0.06	0.08	0.48	0.08	0.48
4D-12	24.10	0.53	0.04	0.21	0.27	0.05	0.07	0.09	0.13	0.04	0.04	0.02	0.37	0.04	0.49
4D-12	24.55	0.71	0.12	0.37	0.58	0.15	0.20	0.19	0.42	0.07	0.05	0.06	0.40	0.07	0.43
4D-12	25.00	2.24	0.54	1.00	1.88	0.54	0.56	0.40	0.73	0.27	0.17	0.38	0.93	0.21	0.88
4D-13	4.55	5.17	1.43	2.14	5.97	1.76	1.83	1.63	3.58	0.45	0.23	0.83	3.46	0.77	5.31
2D-13	24.70	4.58	1.30	2.14	2.42	0.76	0.88	0.25	0.45	0.32	0.19	0.50	0.97	0.27	1.14
4D-12	27.40	0.66	0.20	0.46	0.19	0.07	0.10	0.03	0.06	0.28	0.17	0.62	0.33	0.14	1.38
1D-14	25.10	1.67	0.33	0.67	0.38	0.12	0.12	0.06	0.06	0.41	0.56	1.37	0.65	0.34	0.97

Supplementary Table 10. Concentrations of individual *n*-alkanes for 3 m increments at the thaw front of the subsea permafrost drill cores 4D-13, 2D-13, 4D-12 from the Buor Khaya Bay, and in the incubated samples (n.d.; not detected). Asterisks indicate values below the calculated Limit of Detection; biomarker ratios were considered not analyzed where all data in numerator or denominator were n.d. or below the Limit of Detection. Chain lengths C_{37}-C_{40} were analyzed but not detected.

Core	Depth	C_{15}	C_{16}	C_{17}	C_{18}	C_{19}	C_{20}	C_{21}	C_{22}	C_{23}	C_{24}	C_{25}	C_{26}	C_{27}	C_{28}	C_{29}	C_{30}	C_{31}	C_{32}	C_{33}	C_{34}	C_{35}	C_{36}
	m											µg g^{-1} OC											
4D-13	7.00	9	n.d.	7	n.d.	5	*2	10	*4	17	*4	19	4	29	*3	30	*4	23	n.d.	10	n.d.	n.d.	n.d.
4D-13	7.40	n.d.	n.d.	*8	n.d.	10	3	15	*9	38	n.d.	37	*5	59	n.d.	65	*0	52	n.d.	14	n.d.	n.d.	n.d.
4D-13	7.85	6	n.d.	6	n.d.	13	10	31	24	83	35	126	39	244	47	338	26	330	17	118	*2	34	27
4D-13	8.15	*0	n.d.	9	n.d.	30	n.d.	96	91	275	109	371	99	607	90	748	51	761	35	273	3	49	40
4D-13	8.45	n.d.	n.d.	*10	n.d.	33	34	67	55	154	77	185	58	294	52	421	29	391	24	119	n.d.	44	n.d.
4D-13	8.70	*15	n.d.	20	n.d.	43	44	118	94	328	150	451	130	810	128	1081	80	1111	49	401	10	67	53
4D-13	8.85	21	n.d.	8	*4	19	18	47	39	125	50	169	51	293	50	388	30	363	17	132	*4	29	23
4D-13	8.95	31	n.d.	12	11	18	18	43	35	104	44	154	38	265	47	325	26	307	24	114	*4	43	41
4D-13	9.00	n.d.	n.d.	n.d.	n.d.	n.d.	n.d.	n.d.	n.d.	n.d.	n.d.	n.d.	n.d.	n.d.	n.d.	n.d.	n.d.	n.d.	n.d.	n.d.	n.d.	n.d.	n.d.
4D-13	9.45	n.d.	n.d.	n.d.	n.d.	*1	*2	*1	*3	n.d.	*2	*0	*1	*2	n.d.	n.d.	n.d.	n.d.	n.d.	n.d.	n.d.	n.d.	n.d.
4D-13	9.55	n.d.	n.d.	n.d.	*0	*0	*0	*1	*1	*0	*5	n.d.	*1	*0	*1	*1	*0	*0	*0	n.d.	n.d.	n.d.	n.d.
4D-13	10.00	n.d.	n.d.	n.d.	n.d.	*1	*3	*0	*2	n.d.	*4	*2	*1	n.d.	*4	*4	*4	n.d.	n.d.	n.d.	n.d.	n.d.	n.d.
2D-13	14.25	*6	n.d.	16	81	81	77	125	115	275	120	280	86	331	76	308	44	231	42	93	47	67	n.d.
2D-13	15.35	*8	n.d.	18	82	76	84	237	204	742	297	1040	258	1814	245	1875	140	2036	93	638	72	112	n.d.
2D-13	15.70	*10	n.d.	*7	*4	*4	*1	*4	*6	*5	*15	*6	*7	*14	*20	*11	*16	*23	n.d.	n.d.	n.d.	n.d.	n.d.
2D-13	15.80	n.d.	n.d.	n.d.	n.d.	n.d.	n.d.	n.d.	*0	n.d.	n.d.	n.d.	n.d.	n.d.	n.d.	n.d.	n.d.	n.d.	n.d.	n.d.	n.d.	n.d.	n.d.
2D-13	15.90	n.d.	n.d.	12	86	79	110	242	223	605	242	699	201	1013	181	935	88	1002	82	427	68	102	n.d.
2D-13	16.00	*11	n.d.	17	106	81	124	252	236	685	277	829	241	1280	232	1366	119	1381	88	519	69	104	n.d.
2D-13	16.40	*18	n.d.	35	174	107	130	239	210	686	264	862	222	1403	210	1389	110	1332	91	460	73	116	n.d.
2D-13	16.90	25	n.d.	23	130	107	142	313	290	738	305	803	243	1074	191	951	103	962	77	374	59	93	n.d.
4D-12	22.00	20	n.d.	13	50	50	64	161	150	403	174	510	157	734	141	742	55	867	43	352	39	69	45
4D-12	23.20	21	n.d.	22	92	70	101	187	178	441	192	610	222	871	189	1338	109	1532	66	587	75	130	n.d.
4D-12	23.65	n.d.	n.d.	n.d.	n.d.	*1	*0	*2	*2	*1	*3	*3	*2	*2	*2	*4	*0	n.d.	n.d.	n.d.	n.d.	n.d.	n.d.
4D-12	23.80	n.d.	6	n.d.	n.d.	n.d.	n.d.	n.d.	n.d.	n.d.	n.d.	n.d.	n.d.	n.d.	n.d.	n.d.	n.d.	n.d.	n.d.	n.d.	n.d.	n.d.	n.d.
4D-12	23.90	16	7	15	56	59	70	194	128	393	155	493	147	681	171	1381	127	2096	91	794	27	81	n.d.
4D-12	23.95	23	n.d.	12	62	67	84	250	166	472	203	613	176	866	213	1649	167	2472	108	1028	n.d.	n.d.	n.d.
4D-12	24.10	*3	n.d.	13	67	57	94	216	202	550	236	707	221	1001	188	1039	81	1267	60	546	45	114	n.d.
4D-12	25.00	n.d.	n.d.	9	59	41	78	166	149	420	169	518	166	780	149	751	52	906	43	302	35	n.d.	n.d.
4D-13	4.55	*1	2	2	4	4	5	11	9	25	12	35	15	82	18	91	8	81	6	38	3	10	2
2D-13	24.70	3	5	13	14	25	28	54	50	91	49	101	51	155	40	158	25	158	17	57	7	9	7
4D-12	27.40	n.d.	7	12	8	10	3	9	5	23	4	29	3	41	4	41	5	39	*3	18	3	n.d.	n.d.
1D-14	25.10	7	43	58	63	90	56	84	76	154	68	149	66	166	38	134	32	77	*9	77	4	31	n.d.

Supplementary Table 11. Concentrations of individual *n*-alkanoic acids for 3 m increments at the thaw front of the subsea permafrost drill cores 4D-13, 2D-13, 4D-12 from the Buor Khaya Bay, and in the incubated samples (n.d.; not detected). Asterisks indicate values below the calculated Limit of Detection; biomarker ratios were considered not analyzed where all data in numerator or denominator were n.d. or below the Limit of Detection.

Core	Depth	C_{12}	C_{13}	C_{14}	C_{15}	C_{16}	C_{17}	C_{18}	C_{19}	C_{20}	C_{21}	C_{22}	C_{23}	C_{24}	C_{25}	C_{26}	C_{27}	C_{28}	C_{29}	C_{30}	C_{31}	C_{32}
	m										µg g^{-1} OC											
4D-13	7.00	*12	24	*28	n.d.	*38	*10	*73	n.d.	*14	n.d.	41	n.d.	58	n.d.	n.d.	n.d.	n.d.	n.d.	n.d.	n.d.	n.d.
4D-13	7.40	17	8	*16	*3	*20	*4	n.d.	n.d.	n.d.	10	6	16	14	17	39	n.d.	n.d.	n.d.	n.d.	n.d.	n.d.
4D-13	7.85	30	19	*82	*27	*378	38	*350	8	31	25	141	64	237	33	232	43	185	41	126	n.d.	n.d.
4D-13	8.15	22	13	*37	*26	*147	*22	*119	11	48	23	90	49	141	47	187	47	164	54	256	32	127
4D-13	8.45	19	11	*36	*22	*218	*19	*169	7	32	19	56	36	76	29	72	21	77	n.d.	n.d.	n.d.	n.d.
4D-13	8.70	35	17	*49	*32	*205	*26	*121	14	57	27	103	61	166	52	189	47	170	66	237	n.d.	n.d.
4D-13	8.85	17	13	*32	*15	*40	23	*232	8	51	17	88	35	128	35	141	35	115	21	101	n.d.	n.d.
4D-13	8.95	12	12	*37	*12	n.d.	*18	*201	12	31	14	75	34	126	29	135	30	110	n.d.	89	n.d.	n.d.
4D-13	9.00	*7	5	*27	*16	*135	*19	*150	6	*17	13	36	29	61	22	63	n.d.	65	n.d.	n.d.	n.d.	n.d.
4D-13	9.45	*4	15	*69	*13	*334	*29	*411	13	*11	n.d.	28	n.d.	49	n.d.	70	n.d.	72	n.d.	n.d.	n.d.	n.d.
4D-13	9.55	20	10	*24	34	*104	*16	*53	12	67	27	102	63	146	52	154	46	133	n.d.	161	n.d.	80
4D-13	10.00	23	20	*52	*23	*28	*34	*435	20	62	23	114	50	155	38	119	31	91	n.d.	79	n.d.	n.d.
2D-13	14.25	*1	1	*6	*2	*92	*3	*99	3	10	18	40	26	95	25	89	19	66	13	51	n.d.	n.d.
2D-13	15.35	4	2	*26	*5	*190	*15	*442	3	21	31	102	53	290	64	395	52	301	44	152	n.d.	81
2D-13	15.70	5	2	*48	*11	*598	*21	*556	5	31	51	139	88	365	99	442	78	323	45	230	n.d.	121
2D-13	15.80	3	2	*15	*4	*108	*5	*141	2	22	38	99	60	295	77	410	74	310	39	169	n.d.	106
2D-13	15.90	3	2	*22	*5	*607	*7	*432	3	15	32	84	52	186	52	189	41	151	27	122	42	72
2D-13	16.00	4	2	*41	*7	*371	*20	*529	4	26	31	105	60	271	64	334	57	232	35	147	n.d.	82
2D-13	16.40	3	1	*11	*4	*201	*15	*130	5	22	38	101	57	236	59	202	51	133	40	120	n.d.	n.d.
2D-13	16.90	*2	2	*4	*2	n.d.	1	n.d.	3	11	40	90	70	300	86	416	84	360	n.d.	232	76	128
4D-12	22.00	13	4	*23	14	624	17	*286	4	31	24	126	79	451	96	555	69	359	47	186	47	96
4D-12	23.20	22	7	54	19	*381	28	547	17	71	36	288	128	810	146	754	88	430	50	226	51	118
4D-12	23.65	10	5	43	13	*65	19	*227	11	46	25	162	83	488	96	484	63	294	36	147	37	82
4D-12	23.80	10	6	40	18	198	30	344	29	161	65	1352	440	2926	494	2163	311	1461	222	1491	179	717
4D-12	23.90	10	4	42	17	*109	19	*235	13	91	40	746	264	1715	277	1368	176	894	112	249	96	266
4D-12	23.95	10	3	*27	14	547	25	*278	12	82	37	613	225	1472	227	1063	138	607	85	483	69	202
4D-12	24.10	6	2	*31	7	*164	9	*331	2	19	18	97	61	346	72	415	52	265	35	129	33	59
4D-12	25.00	11	2	30	7	*38	9	233	2	21	15	111	63	376	63	388	45	288	n.d.	139	25	64
4D-13	4.55	27	11	38	21	475	15	327	9	95	26	238	118	357	74	246	38	190	14	68	n.d.	27
2D-13	24.70	40	20	61	25	970	31	688	21	52	22	125	61	209	53	149	39	133	37	125	n.d.	68
4D-12	27.40	54	13	63	*1	1584	47	1337	32	44	26	153	89	296	83	218	67	151	n.d.	123	n.d.	n.d.
1D-14	25.10	n.d.	22	n.d.	n.d.	n.d.	n.d.	n.d.	n.d.	92	n.d.	318	196	741	420	964	439	828	413	1430	n.d.	n.d.

Supplementary Table 12. Concentrations of individual *n*-alkanols, *β*-sitosterol and sitostanol for 3 m increments at the thaw front of the subsea permafrost drill cores 4D-13, 2D-13, 4D-12 from the Buor Khaya Bay, and in the incubated samples (n.d., not detected). Asterisks indicate values below the calculated Limit of Detection; biomarker ratios were considered not analyzed where all data in numerator or denominator were n.d. or below the Limit of Detection.

Core	Depth	C_{14}	C_{15}	C_{16}	C_{17}	C_{18}	C_{19}	C_{20}	C_{21}	C_{22}	C_{23}	C_{24}	C_{25}	C_{26}	C_{27}	C_{28}	C_{29}	C_{30}	C_{31}	C_{32}	C_{33}	*β*-Sitosterol	Sitostanol
	m													µg g^{-1} OC									
4D-13	7.00	5	1	7	n.d.	n.d.	n.d.	7	2	10	4	11	7	44	7	33	n.d.	n.d.	n.d.	n.d.	n.d.	21	9
4D-13	7.40	6	*1	10	*1	n.d.	n.d.	5	*1	11	2	9	4	27	4	18	5	5	n.d.	n.d.	n.d.	*9	n.d.
4D-13	7.85	6	5	16	n.d.	20	3	104	30	228	63	267	92	594	89	516	91	217	46	97	n.d.	271	89
4D-13	8.15	6	5	15	n.d.	38	n.d.	32	24	140	48	159	59	337	51	235	40	80	17	33	n.d.	96	55
4D-13	8.45	*3	3	8	2	n.d.	n.d.	22	8	66	18	92	26	229	24	135	23	49	10	18	n.d.	58	24
4D-13	8.70	5	5	15	2	n.d.	n.d.	49	26	174	50	175	55	330	46	237	35	79	17	30	n.d.	152	55
4D-13	8.85	4	3	9	n.d.	28	3	47	18	117	41	151	58	311	59	272	59	130	31	63	n.d.	101	50
4D-13	8.95	5	3	10	n.d.	6	1	37	15	112	33	152	53	339	54	295	60	128	30	56	n.d.	112	51
4D-13	9.00	n.d.	*1	*4	n.d.	n.d.	n.d.	8	3	30	9	38	14	102	14	69	15	24	n.d.	n.d.	n.d.	26	n.d.
4D-13	9.45	4	2	6	n.d.	n.d.	n.d.	1	5	16	9	27	18	114	18	72	26	37	n.d.	n.d.	n.d.	16	17
4D-13	9.55	3	2	19	n.d.	*9	n.d.	48	18	168	28	136	29	214	25	180	18	47	6	19	n.d.	219	59
4D-13	10.00	6	4	16	n.d.	11	0	68	23	174	47	212	76	514	79	468	93	203	49	93	n.d.	240	79
2D-13	14.25	n.d.	1	1	1	*0	n.d.	4	3	20	6	32	8	73	8	57	6	18	n.d.	8	n.d.	20	11
2D-13	15.35	n.d.	2	4	3	n.d.	*1	16	9	91	28	156	43	415	32	378	20	60	10	27	n.d.	114	68
2D-13	15.70	n.d.	n.d.	n.d.	5	n.d.	n.d.	30	25	166	71	234	90	802	100	470	61	176	31	88	n.d.	96	59
2D-13	15.80	3	2	6	2	9	*2	24	24	127	61	152	72	434	56	244	23	65	10	26	3	114	72
2D-13	15.90	n.d.	*1	n.d.	3	n.d.	n.d.	23	26	143	70	185	77	553	74	301	39	110	19	55	n.d.	95	67
2D-13	16.00	5	2	6	3	8	*2	27	21	119	52	141	61	382	46	215	20	56	8	24	3	118	74
2D-13	16.40	4	2	6	3	12	2	25	13	110	37	154	45	319	34	226	16	57	7	24	2	115	53
2D-13	16.90	2	2	6	2	7	2	16	17	83	42	84	38	154	23	85	9	21	4	8	n.d.	76	56
4D-12	22.00	*6	4	8	3	11	5	50	40	194	76	189	80	624	60	244	26	73	11	31	4	132	109
4D-12	23.20	7	5	10	5	12	7	78	39	223	70	185	79	593	63	242	27	77	12	36	5	181	137
4D-12	23.65	9	6	11	7	17	8	77	44	220	76	183	86	539	69	250	34	75	13	34	4	177	121
4D-12	23.80	7	6	12	8	26	12	302	74	694	108	418	113	543	80	297	52	133	23	59	6	373	187
4D-12	23.90	6	4	8	4	18	6	195	52	447	66	212	57	280	37	132	22	54	9	22	3	331	152
4D-12	23.95	7	5	9	5	14	8	215	59	537	95	322	87	452	61	239	39	109	18	51	4	380	187
4D-12	24.10	9	4	9	3	13	5	43	42	176	72	168	84	657	66	245	30	77	12	36	4	140	104
4D-12	25.00	6	3	10	2	14	3	28	26	116	46	102	46	339	39	151	16	43	7	19	2	115	75
4D-13	4.55	10	4	18	31	16	10	51	15	102	19	95	19	149	11	130	6	24	3	9	1	246	37
2D-13	24.70	27	12	24	32	26	12	76	47	275	74	252	70	222	41	192	22	72	14	26	3	106	73
4D-12	27.40	29	8	14	61	22	32	36	10	106	21	105	21	76	11	61	8	26	3	8	n.d.	52	46
1D-14	25.10	145	38	51	305	51	197	50	21	82	25	106	26	47	8	41	n.d.	30	n.d.	n.d.	n.d.	131	40

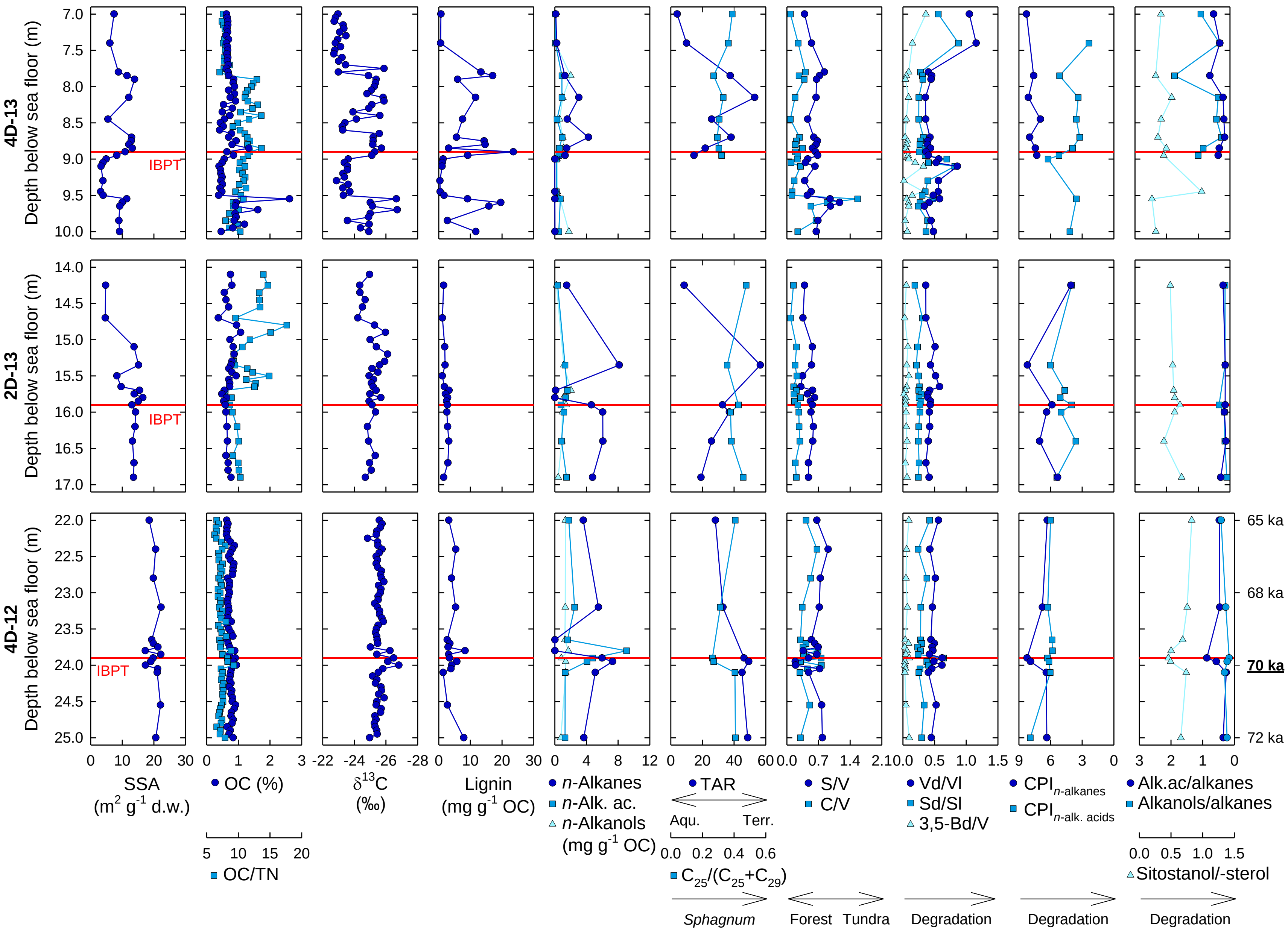
4D-13
Depth below sea floor (m)
2D-13
Depth below sea floor (m)
4D-12
Depth below sea floor (m)
IBPT
65 ka
68 ka
70 ka
72 ka
SSA
(m2 g-1 d.w.)
OC (%)
OC/TN
δ13C
(‰)
Lignin
(mg g-1 OC)
n-Alkanes
n-Alk. ac.
n-Alkanols
(mg g-1 OC)
TAR
Aqu.
Terr.
C25/(C25+C29)
Sphagnum
S/V
C/V
Forest
Tundra
Vd/Vl
Sd/Sl
3,5-Bd/V
Degradation
CPIn-alkanes
CPIn-alk. acids
Degradation
Alk.ac/alkanes
Alkanols/alkanes
Sitostanol/-sterol
Degradation

Supplementary Figure 1. Depth profiles of 3 m sections across the ice-bonded permafrost table (IBPT; red line) of the subsea permafrost cores 4D-13, 2D-13, and 4D-12. Plotted are specific surface area (SSA), total organic carbon (OC), total organic carbon over total nitrogen (OC/TN) and organic carbon $\delta^{13}C$ values, concentrations of lignin phenols, HMW *n*-alkanes, HMW *n*-alkanoic acids, and HMW *n*-alkanols, as well as biomarker proxies for organic matter sources (TAR, terrigenous-aquatic ratio; $C_{25}/(C_{25}+C_{29})$ *n*-alkane ratio indicating *Sphagnum* contribution; S/V, syringyl/vanillyl phenols; C/V, cinnamyl/vanillyl phenols), lignin degradation state (Sd/Sl, syringic acid/syringaldehyde; Vd/Vl, vanillic acid/vanillin; 3,5-Bd/V, 3,5-dihydroxybenzoic acid/vanillyl phenols), and lipid degradation state ($CPI_{n\text{-alkanes}}$, $CPI_{n\text{-alk. acids}}$, carbon preference indices of HMW *n*-alkanes and *n*-alkanoic acids; HMW *n*-alkanoic acids/HMW *n*-alkanes; HMW *n*-alkanes/HMW *n*-alkanes; sitostanol/*β*-sitosterol). Ages based on linear interpolation of optically stimulated luminescence dates are shown for core 4D-12.

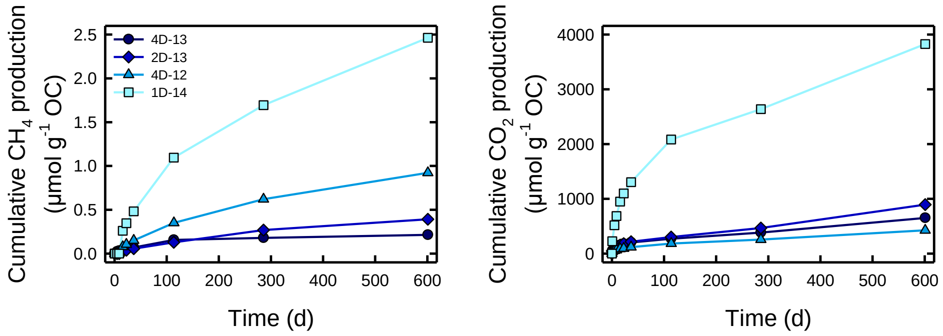

Supplementary Figure 2. Cumulative production of CH_4 and CO_2 by decomposition of thawed subsea permafrost organic matter at 4°C, under anoxic conditions. Samples were from 4.55 m (4D-13), 24.7 m (2D-13), 27.4 m (4D-12) and 25.1 m (1D-14) depth.

References

1. Ankjærgaard, C., Jain, M., Thomsen, K. J. & Murray, A. S. Optimising the separation of quartz and feldspar optically stimulated luminescence using pulsed excitation. *Radiat. Meas.* **45**, 778–785 (2010).

2. Banerjee, D., Murray, A. S., Bøtter-Jensen, L. & Lang, A. Equivalent dose estimation using a single aliquot of polymineral fine grains. *Radiat. Meas.* **33**, 73–94 (2001).

3. Murray, A. S. & Wintle, A. G. Luminescence dating of quartz using an improved single-aliquot regenerative-dose protocol. *Radiat. Meas.* **32**, 57–73 (2000).

4. Murray, A. S. & Wintle, A. G. The single aliquot regenerative dose protocol: Potential for improvements in reliability. *Radiat. Meas.* **37**, 377–381 (2003).

5. Galbraith, R. F. et al. Optical dating of single and multiple grains of quartz from Jinmium rock shelter, Northern Australia: Part I, Experimetal design and statistical models. *Archaeometry* **41**, 339–364 (1999).

6. Murray, A. S., Marten, R., Johnston, A. & Martin, P. Analysis for naturally occurring radionuclides at environmental concentrations by gamma spetrometry. *J. Radioanal. Nucl. Chem.* **115**, 263–288 (1987).

7. Durcan, J. A., King, G. E. & Duller, G. A. T. DRAC: Dose Rate and Age Calculator for trapped charge dating. *Quat. Geochronol.* **28**, 54–61 (2015).

8. Goñi, M. A. & Montgomery, S. Alkaline CuO oxidation with a microwave digestion system: Lignin analyses of geochemical samples. *Anal. Chem.* **72**, 3116–3121 (2000).

9. Vonk, J. E., van Dongen, B. E. & Gustafsson, Ö. Lipid biomarker investigation of the origin and diagenetic state of sub-arctic terrestrial organic matter presently exported into the northern Bothnian Bay. *Mar. Chem.* **112**, 1–10 (2008).

10. Röckmann, T. et al. In situ observations of the isotopic composition of methane at the Cabauw tall tower site. *Atmos. Chem. Phys.* **16**, 10469–10487 (2016).

11. Alvarez, R. & Alvarez, C. R. Soil organic matter pools and their associations with carbon mineralization kinetics. *Soil Sci. Soc. Am. J.* **64**, 184–189 (2000).

12. Shakhova, N. et al. Current rates and mechanisms of subsea permafrost degradation in the East Siberian Arctic Shelf. *Nat. Commun.* **8**, 15872 (2017).

13. Ulyantsev, A. S., Bratskaya, S. Y., Romankevich, E. A., Semiletov, I. P. & Avramenko, V. A. Particle size composition of Holocene–Pleistocene deposits of the Laptev Sea (Buor-Khaya Bay). *Dokl. Earth Sci.* **467**, 241–245 (2016).

14. Blott, S. J. & Pye, K. Gradistat: A grain size distribution and statistics package for the analysis of unconsolidated sediments. *Earth Surf. Process. Landforms* **26**, 1237–1248 (2001).
